# Comparative Evaluation of Cleaning Efficacy of Interdental Brush and Interdental Floss in Orthodontics Patients From Vidarbha Region: An Interventional Study

**DOI:** 10.7759/cureus.46191

**Published:** 2023-09-29

**Authors:** Yukta N Umalkar, Vikrant V Jadhav, Priyanka Paul, Kaushiki P Saoji

**Affiliations:** 1 Orthodontics and Dentofacial Orthopaedics, Sharad Pawar Dental College, Datta Meghe Institute of Higher Education and Research, Wardha, IND; 2 Public Health Dentistry, Sharad Pawar Dental College, Datta Meghe Institute of Higher Education and Research, Wardha, IND

**Keywords:** dental caries, oral health, dental plaque, interdental floss, interdental brush

## Abstract

Background

Plaque and dental caries are the primary agents causing gingival and periodontal diseases, eventually progressing into tooth loss. If oral hygiene practice is poor, plaque easily accumulates on the tooth surface, especially in interproximal areas. To maintain a good oral environment, it is mandatory to remove or at least reduce the percentage of plaque formation from the oral cavity. To achieve this, interdental aids should be used along with toothbrushes, as cleansing the teeth only with a toothbrush is not effective. Various interdental aids, like interdental brushes, floss, toothpicks, etc., are now available on the market. The objective of the current survey was to rate knowledge as well as make a comparison between the cleaning effectiveness of interdental brushes and interdental floss to determine which was better at reducing plaque accumulation and, subsequently, dental caries. The survey was accessed by measuring individual plaque and gingival index before and after using interdental cleaning aids.

Methodology

The objective of the survey was to evaluate and analyze the efficiency of interdental brushes and interdental floss in maintaining oral hygiene among orthodontic patients residing in the Vidarbha region. After receiving approval from the Ethical Committee DMIHER(DU)/IEC/2023/721, a study was conducted over a 30-day period, focusing on a group of 100 individuals aged between 15 and 30 years, and their assessments were analyzed. The patient was briefed about the study and asked to make use of an interdental brush and interdental floss. The gingival index and plaque index were calculated on the same patients before and after the use of the interdental brush and interdental floss to determine which was better at reducing plaque accumulation on the surface of teeth. Descriptive analysis, unpaired for intergroup comparison, and paired T-tests for intragroup comparison were used. The software used was SPSS 24.0 (IBM Corp., Armonk, NY) and GraphPad Prism 7.0 (GraphPad Software, Inc., La Jolla, CA).

Result

Everyone's tooth surfaces naturally develop a thin layer of plaque biofilm, but the presence of heavy plaque deposits on teeth indicates poor dental hygiene, which can lead to various oral health issues. Failure to improve dental hygiene status can result in problems such as halitosis, gingival issues, periodontal disease, and eventually tooth loss. Dentists play a crucial role in raising awareness about these concerns among their patients and providing education on effective oral care practices, including the use of interdental aids in conjunction with toothbrushes. When comparing the effectiveness of interdental brushes and dental floss in removing plaque, interdental brushes have been found to be more efficient. They not only excel in plaque removal but also contribute to a reduction in gingival problems. The statistical analysis supports this, with a significant p-value of less than 0.01 for both the plaque index and gingival index when using interdental brushes, indicating their superior performance in maintaining oral health.

Conclusion

The study will help every individual improve their oral hygiene status with the help of an interdental aid and a toothbrush. This will reduce the chances of having gingival and periodontal diseases and eventually reduce the risk of tooth loss.

## Introduction

Improper tooth alignment may not be life-threatening, but it can have adverse effects on oral tissue health and may contribute to emotional and behavioral issues [1]. Recent research findings have presented varying perspectives on the relationship between dental misalignment and the occurrence of tooth decay. Some studies [2,3] have not definitively established a direct connection between these factors, while contrasting research [4] has hinted at the possibility that individuals with misaligned teeth might experience a reduced risk of tooth decay. The apparent discrepancy observed between the studies with regard to the association between dental misalignment and tooth decay can be attributed to a multitude of factors, including variations in study design, study populations, and measurement methodologies. To gain a more comprehensive and conclusive understanding of this relationship, it is imperative that further research be conducted, taking into account potential mechanisms and confounding variables. This endeavor will contribute to a more comprehensive and definitive elucidation of the connection between dental misalignment and tooth decay.

To address malocclusions, various orthodontic appliances, both removable and fixed, are utilized. However, while these appliances can be beneficial, they may also have associated negative side effects. The use of such appliances can lead to alterations in oral microorganisms [5-8]. In cases where orthodontic treatment is not required and no appliances are worn, oral health outcomes appear to remain similar [9]. Nevertheless, some authors have raised concerns about the potential unexpected consequences of removable appliances on tooth enamel [10,11]. In fixed orthodontic treatments, the placement of orthodontic bands, brackets, ligatures, and other devices on teeth can increase the accumulation of plaque, making it more challenging to maintain good oral hygiene. This, in turn, leads to an elevated bacterial load in the mouth [12,13]. Consequently, the buildup of plaque on both soft and hard oral tissues is recognized as a primary contributor to periodontal disease. Poor dental hygiene maintenance can exacerbate this issue, increasing the likelihood of developing gingivitis, which can then progress to periodontal disease [14,15]. These conditions are facilitated by the presence of acidophilic *Streptococcus mutans* in the plaque [16].

Dental caries can be mitigated through mechanical and chemical removal of bacterial plaque, and there is substantial evidence supporting the hypothesis that improved dental hygiene can lead to reduced gum swelling [17,18]. Periodontitis tends to progress more rapidly in the spaces between teeth [19], prompting the development of various toothbrush models designed to maximize plaque removal [18]. While toothbrushes are effective at removing plaque from tooth surfaces, interdental areas, especially those between molars and premolars, pose a challenge. To maintain oral hygiene in these interdental zones, specific tools are necessary [19]. Several interdental cleaning aids have been devised to address this issue, such as wooden sticks (toothpicks), known for their convenience and simplicity of use. Toothpicks are particularly useful when interdental brushes cannot be utilized due to limited access points. Moreover, they are cost-effective and practical for on-the-go use, as they are disposable, unlike floss or interdental brushes [20,21]. Although many products claim to reduce plaque levels and gingival irritation, they often prove time-consuming, and their effectiveness relies on the skill and expertise of the user [22].

The primary aim of this survey was to assess knowledge and compare the cleaning effectiveness of interdental brushes and interdental floss in reducing plaque accumulation. The study will help in generating valuable, evidence-based data that can significantly enhance clinical practices within the field of dentistry. Also, this can serve as a foundation for dentists to enhance their patient care techniques, ultimately leading to improved patient outcomes. Additionally, it has the potential to exert a considerable influence on the development of guidelines and best practices within the dental community. Consequently, this research endeavor has the capacity to bring about positive change for both dental professionals and their patients by facilitating more effective preventive measures and alleviating the burden of dental diseases.

## Materials and methods

Material required

The materials used for accessing the survey were the interdental brush, interdental floss, mouth mirror, periodontal probe, and dental explorer.

Interdental Brush

**Figure 1:**
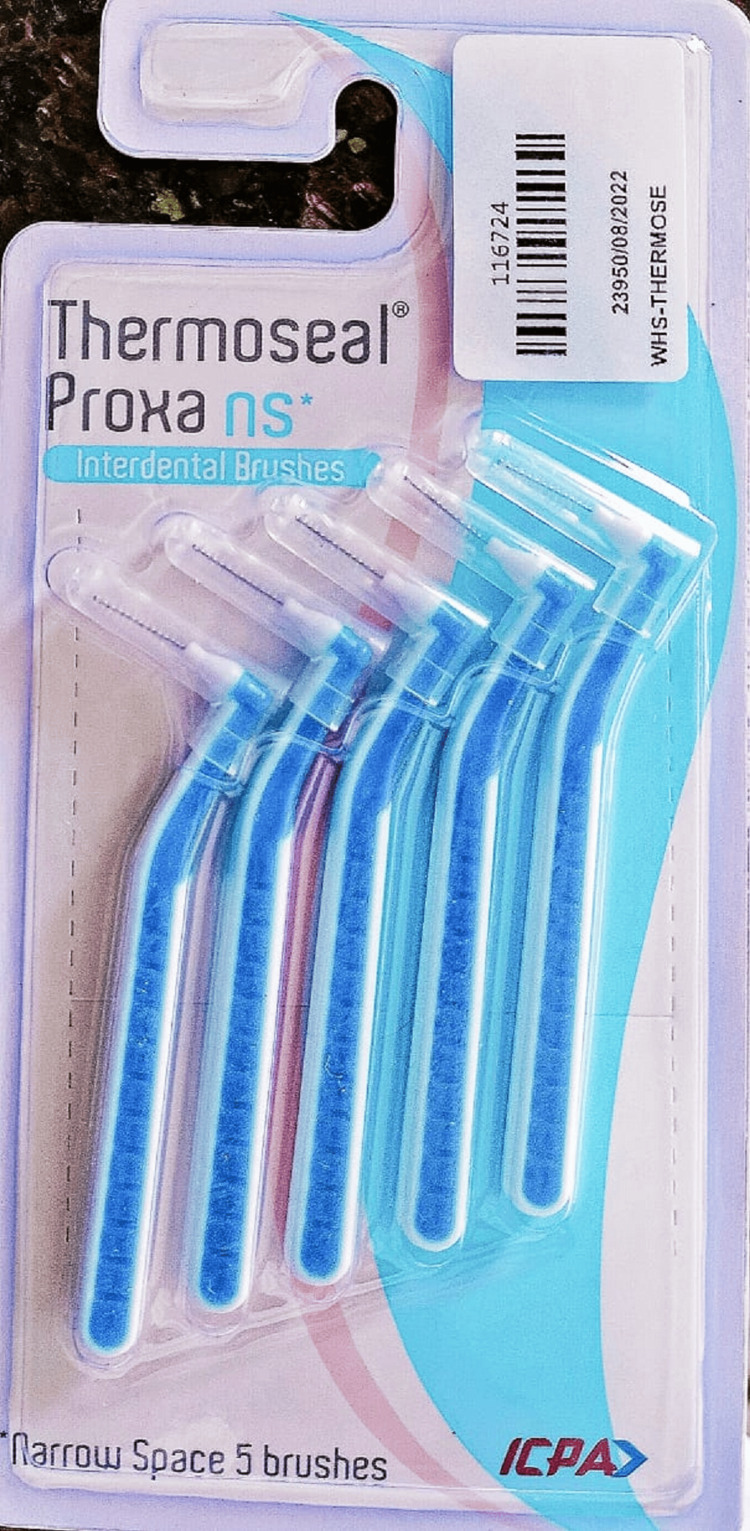
Interdental brush

By removing remaining food particles and plaque in between the teeth, interdental toothbrushes aid in the prevention of gingivitis. They have microbristle tips that easily cleanse the little spaces between the teeth and are available in various measurements. They are of prime importance during orthodontic treatment as they clean the areas that cannot be reached by toothbrushes, and one can buy them easily from medical stores. These brushes are available on the market. The interdental brushes used for the study were Thermoseal Proxa NS (Figure 1).

Interdental Floss

**Figure 2:**
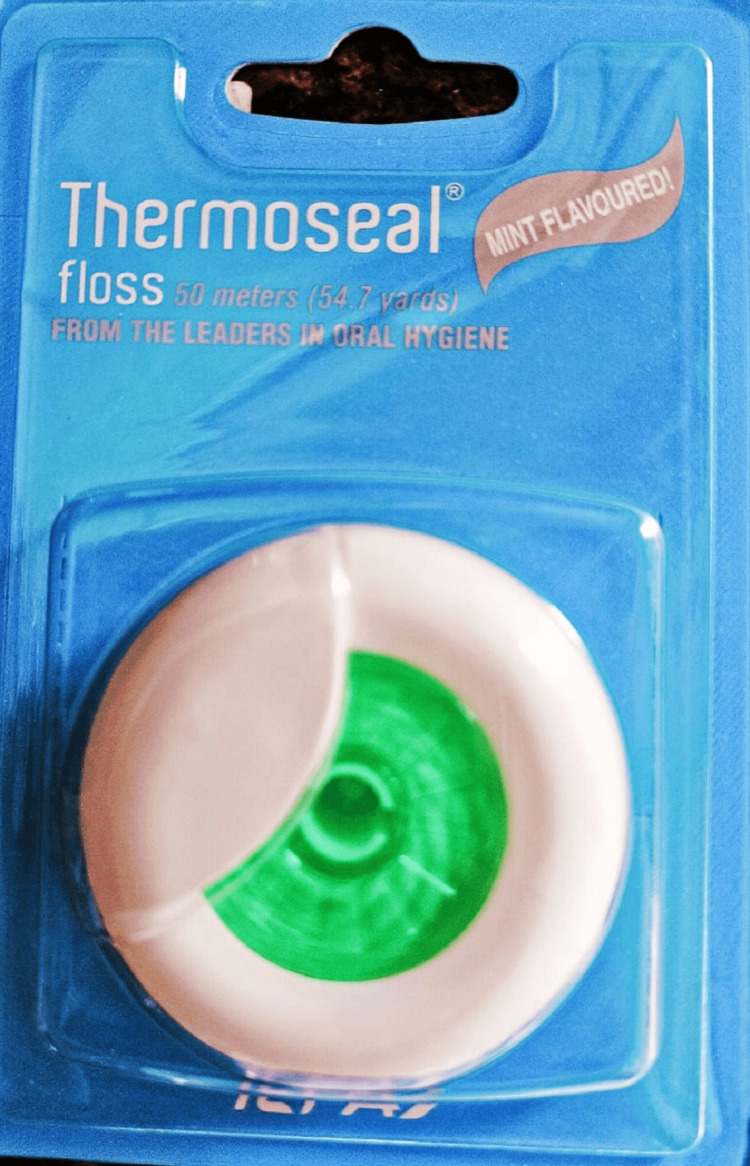
Interdental floss

Dental floss is a string of microfibrils that cleanses the teeth interproximally by eliminating trapped foodstuffs. It should be used on a daily basis to maintain an oral environment that is healthy and disease-free. There are several types of floss, such as multifilaments, waxed monofilaments, and unwaxed monofilaments. Monofilament dental floss that has been wax-coated slips smoothly between teeth but is often more expensive than its uncoated competitors. They are available on the market. The interdental floss used for the study was Thermoseal floss 50 meters (Figure 2).

Methodology

The goal of the survey was to assess and analyze the cleaning effectiveness of interdental brushes as well as interdental floss in orthodontic patients in the Vidarbha region.

Ethical consideration

After receiving approval from the Ethical Committee DMIHER(DU)/IEC/2023/721 of Datta Meghe Institute of Medical Sciences, Deemed University, the study was conducted in the Department of Orthodontics and Dentofacial Orthopaedics at Sharad Pawar Dental College.

Study population

The following prospective interventional study was held on individuals aged 15 to 30 years for 30 days. The assessment was done for 100 people, and the selection criteria for the study population encompass individuals who exhibit elevated plaque levels and experience bleeding gums. High-quality interdental brushes and interdental floss from reputable brands were chosen for the study, with careful consideration to ensure they would not pose any harm to the study population. Patients were explained how to use dental floss and an interdental brush along with their consent, and both of these cleaning aids were randomly distributed among the study population. Patients were evaluated for gingival and plaque index at two intervals, i.e., before the commencement of the study and at the end of three months.

Inclusion and exclusion criteria

Eligibility criteria included patients receiving orthodontic care and patients who are physically fit and can perform flossing and brushing. Exclusion criteria include patients who were done with or not started with orthodontic treatment, physically handicapped patients, and mentally retarded patients.

Sample size

The sample size is determined using the following formula: a sample size of 100, divided into two groups of 50 each, was determined using the formula n = (zα/2)^2 * σ^2/E^2, where σ represents the previously expected value, E stands for the desired margin of error, which was 5%, and zα/2 corresponds to the confidence interval. This sample size was chosen to achieve a statistical power of 80%.

Method

Gingival Index

**Figure 3:**
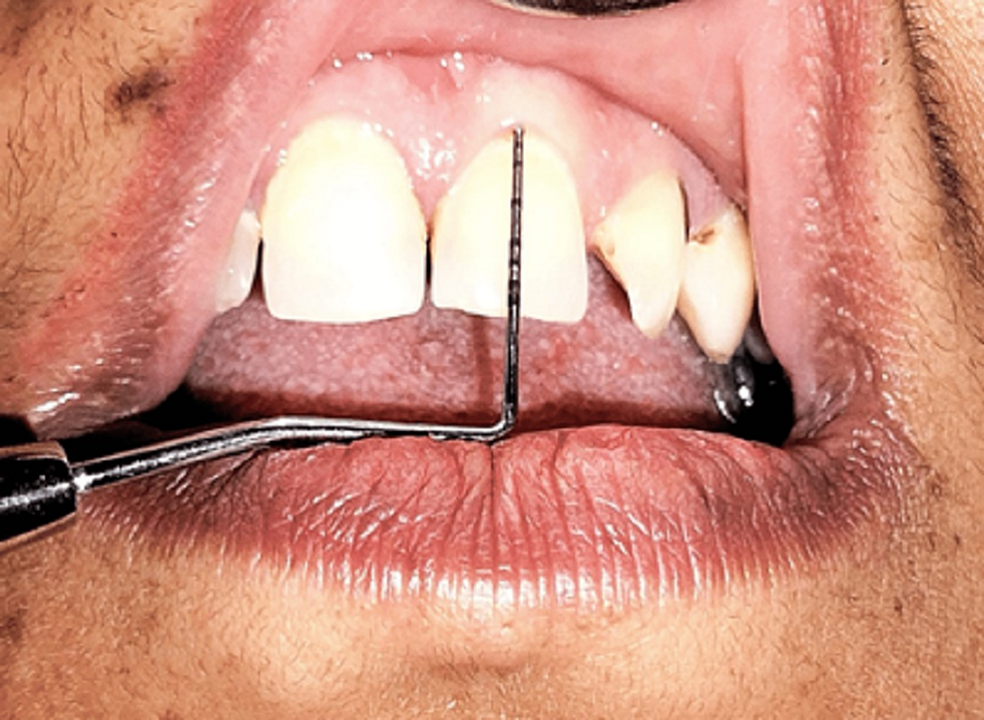
Measuring gingival score

The gingival index was developed by Loe [23] solely to assess the severity of gingivitis. It exhibits good potency, dependability, and usability. Table 1 provides scoring criteria for the calculation of the gingival score, and Table 2 gives an interpretation of the same. Figure 3 shows the measuring gingival score.

**Table 1:** Scoring criteria

Score	Criteria
0	Swelling redness not found/typical gums.
1	Minor edema, color slightly altered, mild irritation, and no blood upon exploring.
2	On exploring, there is mild blood loss, mild edema, mild enlargement, mild swelling, and mild glaze.
3	pronounced erythema and enlargement, prolonged swelling, ulcers, and propensity for continuous bleed.

**Table 2:** Interpretation

Gingival score	Condition
0.1–1.0	Mild gingivitis
1.1–2.0	Moderate gingivitis
2.1–3.0	Severe gingivitis

Plaque Index

**Figure 4:**
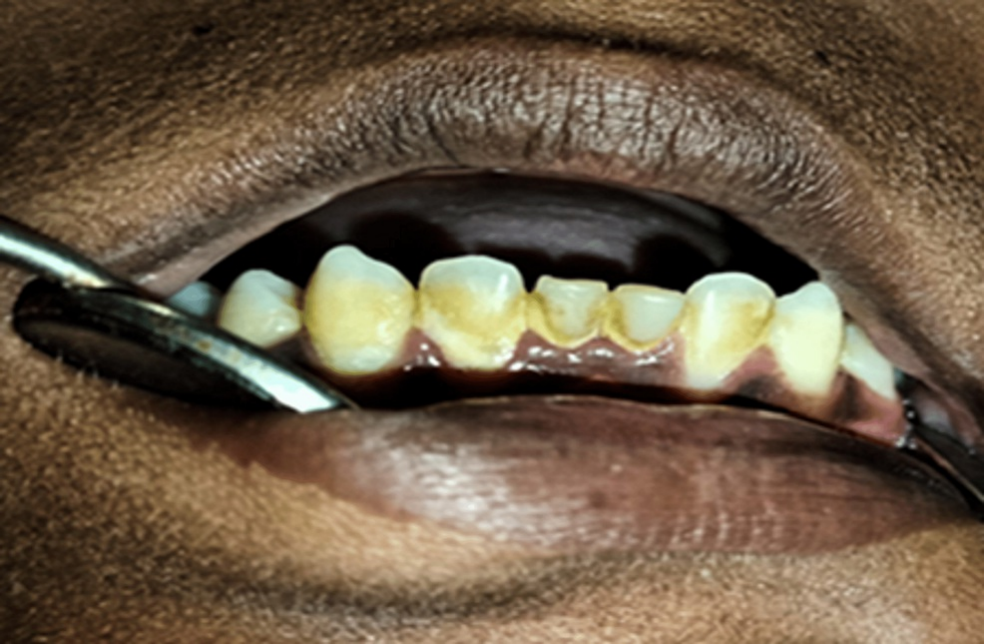
Plaque deposits on the teeth

Loe [23] described the plaque index. It is used to assess plaques and demonstrates good validity and reliability. Table 3 provides scoring criteria for plaque deposits, and Table 4 interprets the same. Figure 4 shows plaque deposits on the teeth.

**Table 3:** Scoring criteria

Score	Criteria
0	Missing plaque’ biofilm.
1	The free gingival edge and the surrounding region of the teeth are covered in a layer of plaques. Only a probe swept along the tooth's surfaces will reveal the plaque.
2	Fine aggregates are visible to the human eye in marginal pockets as well, along the border of gingiva, and on the neighbouring tooth's surfaces.
3	A lot of fine deposits on the adjoining tooth's surfaces, in the occlusal pocket, and along the edge of the gums.

**Table 4:** Interpretation

Plaque score	Condition
0	Excellent
0.1–0.9	Good
1.0–1.9	Fair
2.0–3.0	Poor

Statistical analysis

The statistical analysis employed in this study involved several key methods. Descriptive analysis was utilized to provide a comprehensive overview of the data, while unpaired T-tests were employed for comparing different groups. Additionally, paired T-tests were used for comparing data within the same group. The software tools used for these analyses were SPSS version 24.0 (IBM Corp., Armonk, NY) and GraphPad Prism version 7.0 (GraphPad Software, Inc., La Jolla, CA).

Sample size, represented as "n" in statistics, signifies the quantity of observations or data points gathered from a population or dataset. In hypothesis testing and statistical significance testing, a p-value is a measure of the strength of evidence against a null hypothesis. It quantifies the probability of observing data as extreme as the observed results (or more extreme) if the null hypothesis were true. A low p-value (typically below a significance level like 0.05) indicates that the data provides strong evidence against the null hypothesis.

## Results

Table 5 shows the assessment of the age and gender of study participants using descriptive statistics. In the interdental brush group, most of the study participants were from 15 to 20 years old (28) followed by 21-25 years old (13), whereas in the interdental floss group, out of 50 study participants, most of them were from 21 to 25 years old (21) followed by 26-30 years old (16), where the p-value is 0.0094. This table also shows the gender distribution of study participants in the interdental brush group: 52.0% were males and 48.0% were females, whereas in the case of the interdental floss group, 50% of study participants were males and 50% of study participants were females, and the p-value is 0.8414.

**Table 5:** Assessment of age and gender of study participants using descriptive statistics N: number, %: percentage

Variable	Interdental Brush		Interdental floss		Chi-square	P-value
	N	%	N	%		
Age						
15–20 years	28	56	13	26	9.3302	0.0094
21–25 years	13	26	21	42		
26–30 years	9	18	16	32		
More than 30 years	0	0	0	0		
Gender						
Male	26	52	25	50		
Female	24	48	25	50	0.04	0.8414

Table 6 shows that in the case of the interdental brush group, the pre-use of interdental brush P.I. mean was 2.2700 (SD=0.379), which comes under the poor condition of P.I. In contrast, the post-use shows 1.3520 (SD=0.484), which comes under the fair condition of P.I. from this association. Statistically, a meaningful result was discovered (p≤0.01).

**Table 6:** Association of pre- and post-plaque index and gingival index of interdental brush group using paired ‘T-test’. N: number, SD: standard deviation.

Variables		N (n=100)	Mean	SD	Standard error mean	T-value	P-value
Plaque index	Pre	50	2.2700	0.379	0.053	10.544	<0.01
	Post	50	1.3520	0.484	0.068		
Gingival index	Pre	50	2.3040	0.379	0.053	11.432	<0.01
	Post	50	1.3080	0.485	0.068		

The same result shown by the G.I. in pre-use of interdental brushes was 2.3040 (SD=0.379), which comes under severe gingivitis, whereas the post-use shows 1.3080 (SD=0.485), which comes under moderate gingivitis; from this association, a statistically meaningful result was discovered (p≤0.01).

Table 7 shows that in pre-use of interdental floss, P.I. was 2.5040 (SD=0.412), which comes under the poor condition of P.I. In contrast, post-use also shows 2.2820 (SD 0.415), which comes under this association's poor state of P.I.; the statistically meaningful result was discovered (p=0.009).

**Table 7:** Association of pre n post-plaque index and gingival index of interdental floss group using paired ‘T-test’ N: number, SD: standard deviation, S: significant

Variables		N (n=100)	Mean	SD	Standard error mean	T-value	P-value
Plaque index	Pre	50	2.5040	0.412	0.058	2.681	0.009 (S)
	Post	50	2.2820	0.415	0.058		
Gingival index	Pre	50	2.4540	0.363	0.051	2.979	0.004 (S)
	Post	50	2.2380	0.361	0.051		

The G.I. showed the same result in pre-use of interdental floss: 2.4540 (SD=0.363), which comes under severe gingivitis, whereas post-uses show 2.2380 (SD=0.361), which comes under moderate gingivitis; from this association, the statistically meaningful result was discovered (p=0.004).

## Discussion

Interdental zones, or proximal areas, are considered primary zones that favor plaque retention. To maintain an excellent oral environment, removing or at least reducing the percentage of plaque formation from the oral cavity is mandatory. This will be achieved only by cleansing the teeth with a toothbrush, along with some interdental aids such as an interdental brush and interdental floss. Plaque indices have been used in past studies to compare how well different interdental cleaning methods remove plaque. To measure the effectiveness of oral hygiene products, current studies indicate that the rate of gingival inflammation and bleeding should be decreased rather than plaque elimination alone. The depletion in gum irritation and bleeding reflects the patients' improved ability to remove plaque [24]. This survey was conducted to see how well an innovative interdental hygiene tool prevents the buildup of interdental plaque and gum irritability.

Before the intervention, the patient exhibited elevated plaque levels, an uptick in the occurrence of cavities, and reported bleeding gums, all indicative of deteriorating oral health. However, following the intervention, which involved the implementation of interdental aids such as interdental brushes, there was a noticeable transformation in the oral environment. Plaque levels notably decreased, and there was a marked improvement in the condition of the patient's gums, as bleeding gums were no longer a concern. This suggests that the intervention had a positive impact on the patient's oral health.

These findings carry significant implications for the dental community, offering valuable insights that can empower dental professionals to offer well-informed recommendations to their patients regarding the most effective interdental cleaning techniques for reducing plaque buildup. Such insights hold great potential for enhancing patient outcomes. Furthermore, this research stands to contribute to the establishment of evidence-based guidelines and best practices in the field of dentistry. Dental associations and organizations can leverage this data to formulate evidence-backed recommendations for interdental cleaning, subsequently disseminating this knowledge to dental practitioners worldwide. This approach not only promotes the dissemination of best practices but also holds promise for cost-effectiveness. By identifying the significantly more efficient interdental cleaning method in terms of plaque reduction, patients and healthcare systems may experience cost savings as they can invest in more effective tools and potentially reduce the need for costly dental treatments. Dentists can utilize the results of this research to educate their patients on the critical importance of interdental cleaning, providing evidence-based guidance on the choice of appropriate tools. Informed patients are more likely to adhere to recommended oral hygiene practices, ultimately benefiting their oral health. Additionally, this study lays a solid foundation for further research within the realm of dental hygiene. Future researchers can build upon these findings to explore related topics, such as investigating the long-term effects of interdental cleaning methods on overall oral health or assessing the impact of these methods on specific patient populations.

Studies by Becker et al. [19] have tried to contrast the effectiveness of a recently introduced interproximal cleaning aid (Brush Pick) in a split-mouth randomized clinical trial. It has been resoundingly proven that interproximal regions are the areas where periodontal disease is most prevalent and intense, and it also progresses faster interdentally [19]. Therefore, it is crucial to have appropriate plaque management in these locations. Different toothbrush models have been created to remove as much plaque as possible [18]. According to this study, interdental toothbrushes are more effective at removing plaque than dental floss.

Nyman et al. [25] published a review of various interdental cleaning aids and their efficacy in maintaining oral hygiene. The plaque should be controlled to achieve good results after any periodontal therapy [25]. Therefore, the study concludes that, along with tooth brushing, interdental cleaning aids should also be practiced to minimize plaque accumulation. Plaque management must be optimized for surgical and non-surgical periodontal treatment to be effective. This cannot be accomplished with brushing; supplementary interdental brushing instruments are required. Additional advantages include patient acceptability and simplicity of use. This investigation aims to present a summary of several interproximal cleaning tools and examine the literature to see whether there is agreement about their efficacy. Wooden interdental tools could lessen gingival bleeding, but they do not remove plaque more effectively than brushing [26]. Oral irrigators are a potential technique for lowering gingival inflammation, although there has not been much difference in removing plaque. Oral irrigators and interdental brushes work better for cleaning around dental implants than floss does.

According to Marsh [27], a prevalent problematic oral condition that damages the teeth tissues that support them is periodontal disease or gingivitis. If the condition is not treated, then the tooth can be lost. Research studies demonstrated that dental hygiene would lessen such problems by eliminating bacterial plaque from all areas [27]. A descriptive study was conducted among people using a questionnaire to determine their awareness of interdental cleaning aids and their implementation among the people of Riyadh, Saudi Arabia. Understanding and use of interdental aids among the residents of Riyadh showed that almost the entire population was aware of them, but only a few people implemented them.

Limitations

**Figure 5:**
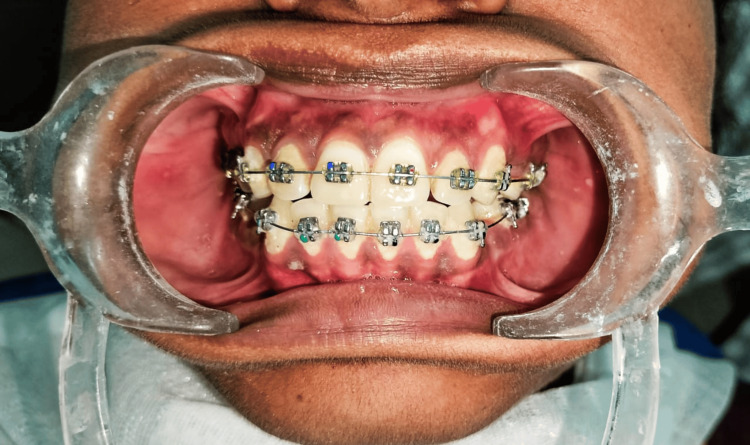
Patient receiving orthodontic care

The current survey focuses on short-term outcomes. It exclusively compares the effectiveness of interproximal brushes and interdental floss without considering other interdental aids. Furthermore, the survey specifically targets a particular age group of patients, exclusively those undergoing orthodontic treatment (Figure 5).

## Conclusions

Interdental cleansing tools play a crucial role in maintaining optimal gingival health and promoting oral hygiene. Research findings suggest that using interdental brushes alongside regular toothbrushing offers several advantages compared to relying solely on a toothbrush. Upon analyzing the presented data, in comparison to traditional dental floss, interdental brushes have demonstrated notably superior effectiveness in reducing both gingival inflammation, with an approximate reduction of 43.26%, and plaque buildup, with an approximately 40.43% reduction. Additionally, they provide the added benefit of increased patient convenience and acceptability. This is particularly important, as patient compliance and comfort are essential factors in maintaining consistent oral hygiene practices.

In conclusion, while interdental brushes offer advantages over traditional flossing, it is essential to emphasize the importance of proper technique and individual patient needs when recommending oral hygiene tools and practices.
